# Respiratory obstruction due to tonsillar lymphoglandular polyp in a brachycephalic dog: a case report

**DOI:** 10.1186/s12917-021-03082-7

**Published:** 2021-12-04

**Authors:** Leah Gabriel, Yazdan Aryazand, Nicole Buote

**Affiliations:** 1 VCA West Los Angeles Animal Hospital, Surgery Department, Los Angeles, California USA; 2 Cornell University College of Veterinary Medicine, Small Animal Surgery Department, 930 Campus Road, Ithaca, NY 14853 USA

**Keywords:** Brachycephalic, BOAS, Lymphoglandular polyps, Tonsillectomy, Tonsil

## Abstract

**Background:**

Respiratory distress is one of the most common afflictions of brachycephalic dogs. Dogs in respiratory distress usually present to the emergency service with a constellation of clinical signs including but not limited to: stertorous breathing, dyspnea, gagging, cyanotic mucus membranes, hyperthermia, and commonly a history of gastrointestinal signs. While Brachycephalic Obstructive Airway Syndrome is the most common cause of respiratory distress in dogs with brachycephalic conformation, any condition eliciting an inflammatory response in the oropharynx, can result in obstruction. There is no previous report of respiratory obstruction leading to emergency tonsillectomy caused by tonsillar polyps.

**Case presentation:**

A 9-month-old male intact English bulldog presented to the emergency service in severe respiratory distress. Due to continued severe dyspnea and cyanosis the patient was induced with propofol (Propofol, Hospira) 4 mg/kg intravenously titrated to effect and tracheal intubation performed. Intubation was noted to be difficult due the presence of two, large, inflamed masses in the oropharynx region. The remainder of his physical exam was unremarkable. Minimum database blood work and chest radiographs revealed only minor abnormalities. The patient was placed under anesthesia and the masses were transected sharply using a carbon dioxide (CO2) laser (Aesculight, Bothell, WA, USA). Anesthesia and recovery were uneventful, and the patient was discharged the following day. Histopathology results of the masses revealed them to be benign lymphoglandular polyps.

**Conclusions:**

This is the first report of bilateral tonsillar polyps causing life-threatening respiratory obstruction in a dog. Both masses were excised safely and completely with the CO2 laser. Difficulties inherent to oropharyngeal surgery include the hemorrhage, small working space, tissue swelling and difficult visualization. Surgical excision of these polyps alleviated all emergent and chronic clinical signs, and the patient’s remains healthy 12-months post-treatment.

## Background

Brachycephalic obstructive airway syndrome (BOAS) is the most common cause of respiratory distress in brachycephalic breeds [1–3]. Dogs in respiratory distress usually present to the emergency service with a constellation of clinical signs including but not limited to: stertorous breathing, dyspnea, gagging, cyanotic mucus membranes, hyperthermia, and commonly a history of gastrointestinal signs. BOAS consists of both primary anatomic abnormalities with which a patient is born, and secondary acquired changes that develop due to subatmospheric and turbulent airway pressures [2, 3]. Primary changes include stenotic nares, elongated soft palate, redundant pharyngeal folds, hypoplastic trachea and aberrant nasopharyngeal turbinates [3]. Secondary changes include everted laryngeal saccules, laryngeal collapse, tracheal and bronchial collapse, tonsillar eversion and gastrointestinal signs (gastro-oesophageal reflux disease and regurgitation). While BOAS is the most common cause of respiratory distress in these patients, any condition eliciting an inflammatory response in the oropharynx, could result in obstruction. The second most common cause affecting dogs younger than 1 year of age with similar clinical signs would be tonsillitis followed by trauma, foreign bodies, and neoplasia [1]. Findings on oral exam in patients suffering from tonsillitis consist of red, friable, and enlarged tonsils without any other discernible obstructive tissue. Secondary bacterial infections are common and cultured bacteria from an inflamed tonsil is usually no different from normal flora of otherwise healthy dogs [4]. Tonsillitis is usually treated medically, with tonsillectomy reserved as a procedure for recurrent cases. Tonsillar polyps are a rare condition in dogs and are most commonly seen in older patients and found incidentally as the majority of dogs are asymptomatic according to two previous studies [5, 6].

To the authors’ knowledge, there is no previous report of respiratory obstruction leading to emergency tonsillectomy caused by tonsillar polyps in a juvenile dog.

## Case presentation

A 9-month-old male intact English bulldog presented to the emergency service in severe respiratory distress. The patient had begun breathing with increased effort over the 12–16 h preceding presentation. Upon presentation, the owner reported that the patient had been regurgitating and vomiting almost daily over the past 3 months, most prominently after a meal. The owner chose not to pursue veterinary care for these regurgitation episodes. Over the previous 3 weeks, the patient had a minimum of four collapsing episodes that lasted anywhere from 20 to 60 s. The patient would always recover fully from these episodes and was back to normal within a few minutes.

**Fig. 1:**
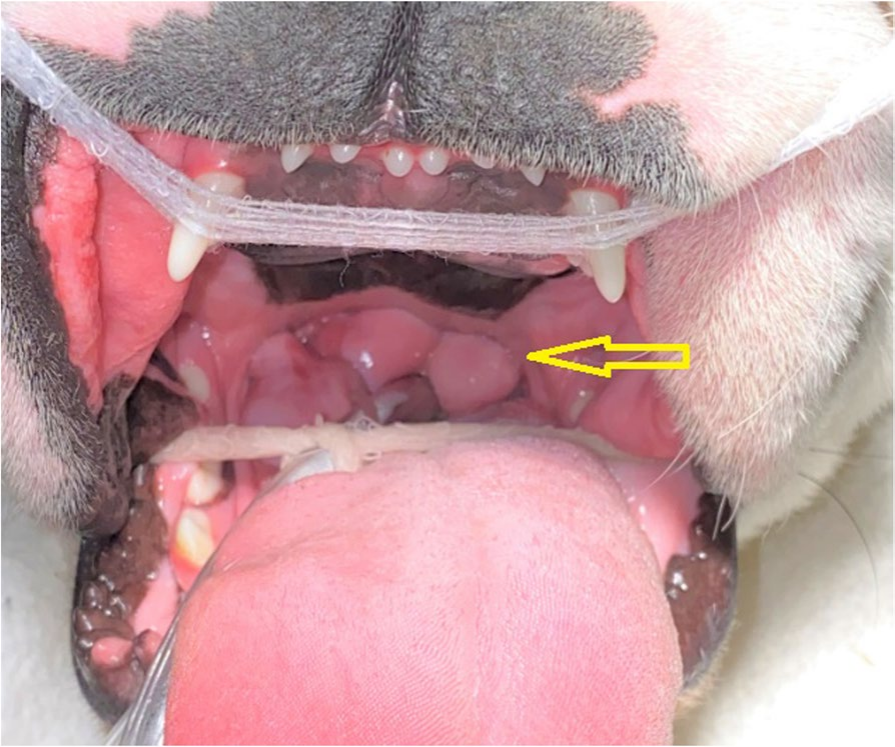
Patient intubated with large tonsillar masses (yellow arrow) covering a majority of the airway in situ

Upon triage, an intravenous catheter was placed, and the patient was given 0.05 mg/kg acepromazine along with 0.2 mg/kg of butorphanol (Torbugesic, Zoetis) intravenously (IV). Due to continued severe dyspnea and cyanosis the patient was induced with propofol (Propofol, Hospira) 4 mg/kg intravenously titrated to effect and tracheal intubation performed. Intubation was noted to be difficult due the presence of two, large, inflamed masses in the oropharynx region (Fig. 1). These masses were causing complete blockage of the airway and they had to be manually retracted to intubate the trachea. The masses were asymmetrical with the right being larger than the left. The patient was placed on 100% oxygen and continued to breath spontaneously. Upon auscultation of his lungs no crackles or wheezes were appreciated but loud referred upper airway sounds were auscultated. The remainder of his physical exam was unremarkable. After intubation, patient was given 0.1 mg/kg dexamethasone (Dexamethasone-SP, VetOne) and 1 mg/kg maropitant (Cerenia; Zoetis) intravenously and a 200 mL Lactated Ringer Solution (LRS, Hospira) IV bolus.

**Fig. 2:**
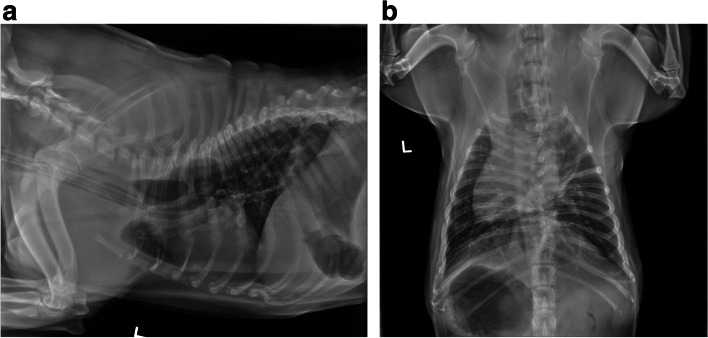
**a** and **b** Left lateral and ventrodorsal radiographic projection of the thorax. There is an endotracheal tube present as well as evidence of esophageal dilatation cranial to the carina with gas accumulation. The pulmonary vasculature and parenchyma are unremarkable

**Fig. 3:**
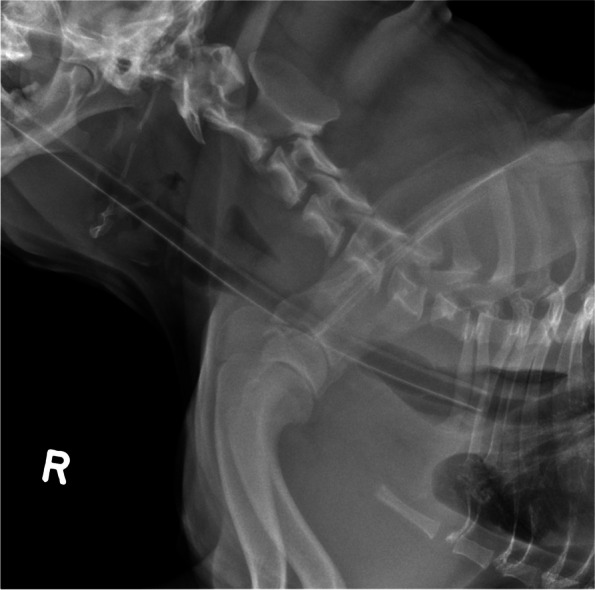
Lateral cervical radiographic projection with evidence of pharyngeal thickening likely secondary to inflammation or edema

An intravenous blood gas (i-STAT, Abbott) sample was obtained at the time of intubation which revealed an elevated blood urea nitrogen 32 (10–26 mg/dL), an elevated Creatinine 1.4 (0.5–1.3 mg/dL) and a decreased TCO_2_ 26 mmol/L (35–45 mmol/L). All other values (Na^+^, K^+^, Cl^−^, Glu, HCT, Hb, Anion Gap) were within normal limits. Three-view thoracic radiographs, and a lateral projection of the cervical region, were performed while the patient remained intubated (Figs. 2 and 3). Radiographs revealed severe generalized esophageal dilatation cranial to the carina with gas accumulation (Fig. 2a, b). The cardiac silhouette was ventrally deviated due to suspect esophageal pathology. Increased soft tissue opacity in the pharyngeal region with probable pharyngeal thickening was noted (Fig. 3). Possible persistent right aortic arch anomaly pathology was discussed with owners, but due to financial limitations, owners declined computed tomography with angiogram and elected to move forward with treatment of the immediate life-threatening upper airway obstruction.

**Fig. 4:**
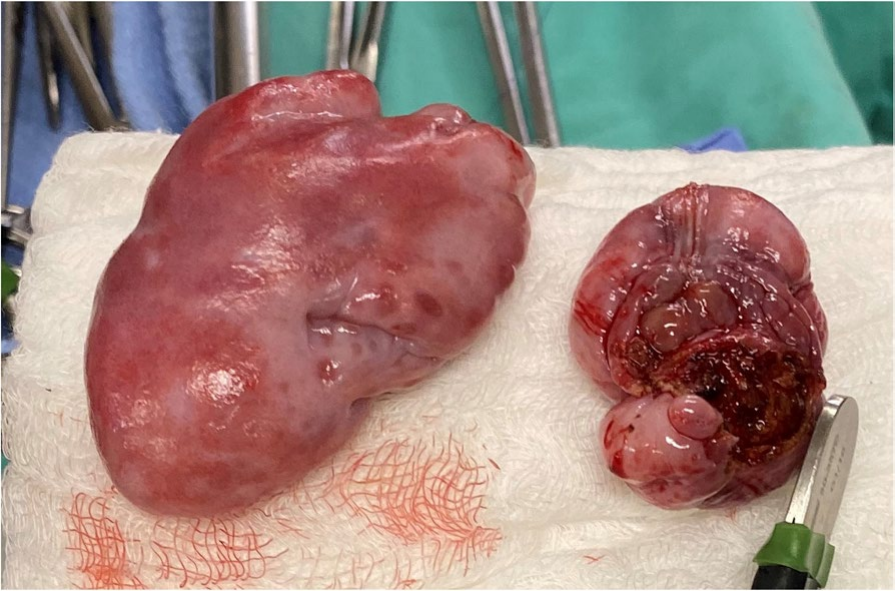
Lymphoglandular masses after curative excisional biopsies. The right sided mass measured 8 cm × 5 cm, the left measured approximately 5 cm × 3 cm

To alleviate concern for congenital cardiac disease before pursuing surgery an echocardiogram was performed. This revealed mild mitral and tricuspid valve dysplasia without atrial enlargement. The patient was placed on inhalant anesthetics (isoflurane) and emergency surgery performed where two, large, soft tissue masses were visualized on either side of the oropharynx (suspect enlarged tonsils). The masses were grasped with long Debakey forceps and transected sharply using a right-angle tip carbon dioxide (CO_2_) laser by cutting in a lateral to medial direction (Aesculight, Bothell, WA, USA). The right sided mass measured 8 cm × 5 cm, with the left measuring approximately 5 cm × 3 cm. (Fig. 4). A classic staphylectomy was also performed using the CO_2_ laser. The patient had moderately everted laryngeal saccules that were sharply excised using Metzenbaum scissors. The patient had normal nares which did not require surgical correction. Anesthesia and recovery were uneventful. The patient recovered in oxygen for 2 h postoperatively was hospitalized overnight on intravenous fluids with metoclopramide (Reglan injection, Baxter Healthcare Corp) 2 mg/kg/day and buprenorphine (Buprenex, Reckitt Benckiser Healthcare) 0.015 mg/kg intravenously every 6 h. The masses were submitted for histopathology and an aerobic culture of the center of the larger mass was also submitted. The patient was offered small balls of wet food the next morning and ate well with no regurgitation. The patient was discharged 24 h post-operatively with trazodone (Trazodone Hydrochloride, TEVA) 6.6 mg/kg by mouth as needed, famotidine (Pepcid, Wockhardt) 0.7 mg/kg by mouth every 12 h for 7 days, and maropitant 2 mg/kg by mouth every 24 h for 4 days.

**Fig. 5:**
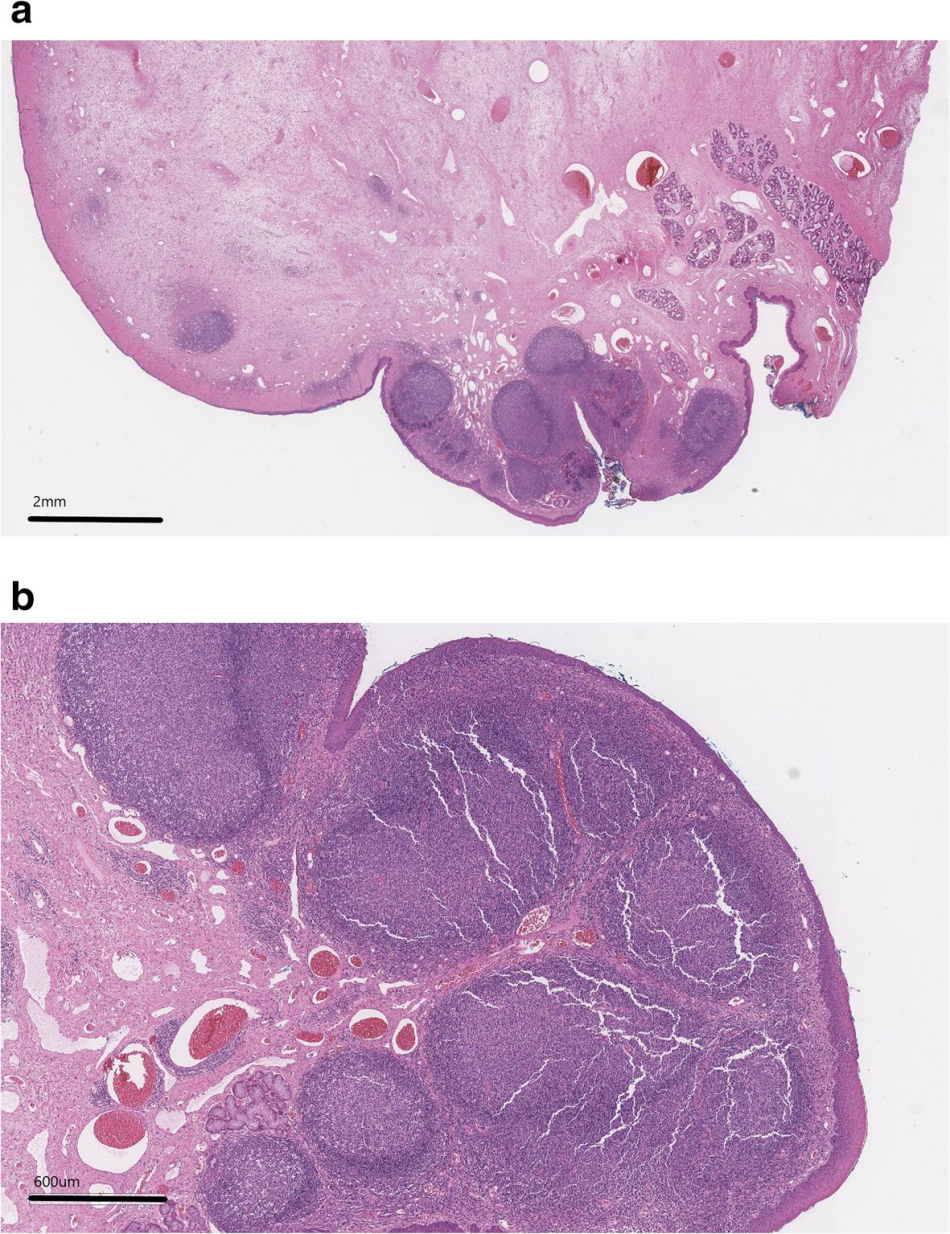
**a** and **b** Photomicrographs of a histopathology slide (a.1X magnification, b. 4X magnification, H&E stain)- polypoid projections of edematous collagen are seen with scattered congested blood vessels with a surface lined by thick squamous epithelium

Histopathology results of the masses revealed complete excision of polypoid projections of edematous collagen with scattered congested blood vessels within them. Several lymphoid aggregates were noted within superficial portions of the projection with scattered glands and ducts (Fig. 5). The surface was lined by thick squamous epithelium. Both masses were found to be benign polyps. The culture revealed *Escherichia coli* so the patient was treated with 10 days of marbofloxacin (Zeniquin, Zoetis) 2.5 mg/kg by mouth once daily based on susceptibility testing.

Follow up appointments were conducted at 14 days and 6 months postoperatively and the patient was noted to be comfortable and asymptomatic with no reports of difficulty breathing or abnormal gastrointestinal signs.

## Discussion & Conclusion

To the authors knowledge, this is the first report of bilateral tonsillar polyps causing respiratory obstruction in a juvenile dog. Tonsillar polyps are rare in both people and small animals and are usually seen during intubation incidentally in older patients undergoing an anesthetic event or during an annual exam [5–9]. Tumors of the tonsil, both benign and malignant are also uncommon in both veterinary and human medicine and almost always are seen in older patients [8, 9]. Two previous studies on tonsillar polyps reported an average age at diagnosis of 9–12.5 years old with a range of 3–15 years. These case series also report the polyps were found incidentally in 71–75% of cases [5, 6]. As BOAS is the most common cause of respiratory distress in brachycephalic patients the finding of oropharyngeal masses in this young dog was surprising. There is one other published report of an upper airway obstruction caused by a polyp in a young patient. In that case a 9-week-old puppy was diagnosed with a nasopharyngeal polyp causing airway obstruction via nasopharyngoscopy [10]. There has only been one report of incidental nonobstructive lymphangiomatosis polyp in an older dog [8]. Polyps are considered a type of benign hyperplasia which we can lead to life-threatening respiratory distress depending on location. The exact pathogenesis of tonsillar polyp formation remains unknown but one theory proposes that chronic inflammation with possible obstructions of the lymphatic channels leads to these changes as described in human medicine [6, 11]. Secondary inflammation may have been present in our patient due to episodes of regurgitation and could be one cause of hyperplasia in this patient. Another possible etiopathogenesis of the tonsillitis in this case could be the coliform infection of the tonsillar tissue but we feel this is most likely secondary to the inflammation from prolonged increased upper airway pressures. It is probable that bacteria were introduced into inflamed tonsillar tissue during the frequent episodes of regurgitation and vomiting reported by the owners. Other authors have suggested hamartomatous proliferation as a likely etiology in people [9].

Surgical excision of the masses and concurrent staphylectomy/sacculectomy allowed for a rapid recovery in this patient and the tonsillectomy proved to be diagnostic as well as therapeutic. Both masses were excised safely and completely with the CO_2_ laser. The use of CO_2_ laser for staphylectomy has been reported previously and been shown to be safe [12]. The most common surgical complication in human medicine following tonsillectomy is bleeding and this can be fatal. Hemorrhage is seen in approximately 5.21% of cases [13] and challenges in controlling bleeding are attributed to the small working space of the oropharynx, tissue swelling and difficult visualization. In veterinary medicine, complications associated with tonsillectomy are rare but include bleeding, swelling, inflammation, prolonged surgical site healing, necrosis or burns to the surrounding tissue, and aspiration of blood or saliva [1]. Veterinarians perform the different aspect of BOAS surgery by various methods, many without the use of a laser, tonsillectomy, however, may benefit from some type of cautery or hemostatic instrument and the possibility of postoperative bleeding should be discussed with owners. Other studies have utilized bipolar sealing devices for tonsillectomy with excellent results but the CO2 laser with right angle tip utilized in this patient allowed for precise and rapid transection and hemostasis with minimal instrumentation in the oropharynx [14, 15]. A recent systematic review on laser tonsillectomy in humans reported that laser techniques were superior regarding intraoperative bleeding and procedure duration and provided equivocal or superior outcomes regarding postoperative hemorrhage, pain, and total healing time [16]. In our case, the signs resolved after removal of the masses and correction of the BOAS and no postoperative complications were encountered. Our patient recovered smoothly from the procedure and on a six-month post-operative phone follow-up, the pet was doing well at home without any respiratory or gastrointestinal signs.

To the authors’ knowledge, this is the first report of an upper airway obstruction in a juvenile dog secondary to bilateral tonsillar polyps. Surgical excision alleviated all clinical signs, and the patient’s preoperative respiratory and gastrointestinal signs have not recurred 12-month post-treatment.

## Data Availability

All data generated or analyzed during this study are included in this published article.

## Abbreviations

BOAS: Brachycephalic Obstructive Airway Syndrome
TCO_2_: Total Carbon Dioxide
CO_2_: Carbon Dioxide
